# A Letter from Denver in Which Its Author Claims to Have Preceded Dr. Emmet in Laceration of the Cervix Uteri as a Cause of Disease

**Published:** 1879-04

**Authors:** W. H. Newman

**Affiliations:** Denver, Col.


### ARTICLE XVI.

A LETTER FROM DENVER, IN WHICH ITS AUTHOR CLAIMS TO HAVE PRECEDED DR. EMMET IN LACERATION OF THE CERVIX UTERI AS A CAUSE OF DISEASE. Read by Dr. W. H. Byford before the Chicago Gynæcological Society, Jan. 31, 1879.

*Denver, Col., Jan.*, 1879.

About four years ago the subject of laceration of the cervix uteri was brought before the medical society of the county of New York, by Dr. T. Addis Emmet, in a paper read before that society, and afterward published in the *American Journal of Obstetrics*, for November, 1874. That paper was received with applause by the New York society, and has since been received with great favor by the profession at large. It would appear from the manner in which the paper has been commented upon, that up to that time the importance of laceration of the cervix had not been at all appreciated by any part of the medical profession, and that except by a few physicians in and about the Woman's Hospital, this lesion had not been discovered. The title of Dr. Emmet's paper, the character of the discussion that took place at the time it was presented, as well as the tone of the publications of other physicians throughout the country, all tend to show that, up to the appearance of that paper, this injury of the cervix was unappreciated, if not unknown.

It is true, that in the *American Journal of Obstetrics* for February, 1869, Dr. Emmet had himself alluded, in a paper on the Surgery of the Cervix, to lateral division or laceration as a condition that sometimes complicated the treatment of dysmenorrhœa or sterility. That paper is entitled, "Surgery of the Cervix in Connection with the Treatment of Certain Uterine Diseases," and is devoted to the treatment of dysmenorrhœa and sterility by division of the cervix. He had not, it would seem, at the time of writing that paper, regarded lateral laceration as a cause of disease, but simply as a lesion that interfered with his method of treating dysmenorrhœa and sterility by anterior or posterior division. But on the 28th of September, 1874, the date of the reading of his paper before the Medical Society of the County of New York, he, for the first time, described this lesion as a cause of uterine disease. In the title of his paper he calls it an unrecognized cause of disease.

Dr. Emmet did not, of course, mean that up to that time he had not recognized laceration, or that Dr. Sims and a few others had not; but what he does say or imply is, that except by a few physicians about the Woman's Hospital, this lesion had not been recognized. All the credit is therefore awarded, by the profession, to Dr. Emmet, for being the first to appreciate and describe the lesion as a cause of disease.

Three years before Dr. Emmet's paper appeared, I had written a paper on this subject, which was intended to demonstrate the importance of this accident. This communication of mine was published in the *American Practitioner* for June, 1871, and was at the time sent, in pamphlet form, to all the leading gynæcologists of the United States. That paper not only shows that I had recognized laceration as a cause, and a very important cause, of disease, but it brought, for the first time, this matter to the attention of the profession.

Besides the two papers of Dr. Emmet already referred to, he has still another, written to explain more fully his mode of treatment. This paper is published in the *American Practitioner* for January, 1877. The reason for publishing it is thus stated by himself: "But, unfortunately, too much has been expected of the operation; it has been performed frequently without understanding its principles, and generally without proper preparatory treatment. The consequence has been that within the circle of my professional acquaintance I have already heard expressions of disappointment from not getting the good results which had been promised for the operation."

This operation consists in making fresh or raw the edges of the rupture, and uniting them with silver wire sutures. That disappointment should have been experienced in a large number of cases treated this way is not, in my judgment, to be greatly wondered at. The condition of the cervix and the mucous membrane covering the cervix is, in a large number of the cases presented for treatment, such as almost to preclude the hope of success. These conditions are well described by Dr. Emmet. The cervix is inflamed, indurated and enlarged. The mucous membrane is inflamed, and its follicles, along the tract of the canal, are in a state of cystic degeneration. To bring together these edges, with the cervix in this diseased state, and expect them to unite, must, it seems to me, very frequently result in disappointment. Dr. Emmet explains the cause of the disappointment in his last article on the subject. He says there must, in many cases, be some preparatory treatment; that when the cervix or its mucous membrane is much diseased, its condition must be improved before the operation is undertaken—the inflammation and hypertrophy must be reduced, and the degenerated follicles removed from the mucous membrane. But just here is the difficulty that so many surgeons have encountered, and will always encounter, in the management of these cases. To remove chronic inflammation and hypertrophy of the cervix, especially if this hypertrophy is due, as he suggests, to arrest of involution, is a work that cannot be accomplished in a few weeks or in a few months. It is always a tedious work, and in not a few cases, either from the exhausted state of the patient, the unfavorable circumstances in which she is situated, or from the extent of disease present, it is a work that cannot be accomplished at all.

In my paper, published more than three years before Dr. Emmet's, I say: "I can see no reason why, after the inflammation is somewhat reduced, and the edges of the wound are fit for union, one or more silver wire sutures may not be used to close the edges, just as Jobert recommended for vesico-uterine fistula."

Dr. Emmet very properly insists that the operation for uniting these edges should not be performed until the parts are fit to be united. This is just what I stated more than three years before his paper was written. This operation had, at my suggestion, been performed by Dr. E. C. Gehrung, formerly of this city, nearly two years before Dr. Emmet's paper was published, and the case described by me in my report on gynæcology to the Colorado State Medical Society in 1875. In this case there was no induration, no hypertrophy, no cystic degeneration. The parts were "fit to be united," and the operation was entirely successful. At the date of the writing of my paper, I had not myself attempted to cure these cases, except the severest ones, and these I had cured by amputation. But at the time Dr. Emmet first called the attention of the profession to laceration of the cervix as an unrecognized cause of disease, I was treating and curing these cases by amputation when the cervix was much diseased, or by sutures when the parts were in condition, or could be brought in condition to unite, and my views had been published and widely disseminated.

But even if the operation of repairing the cervix could always be as successfully performed as it is said to have been in the skillful hands of Dr. Emmet, I still think there are objections to it, for however successfully the union may be effected, the cervix must be left in a mutilated condition. The delicate mucous membrane formerly lining the canal will have been destroyed by the pressure and friction to which it has been subjected, if not by the cystic degeneration it will have undergone. Important changes will also have occurred in the form of the cervix; its conicalness is increased, its integrity impaired.

Dr. Emmet is of opinion that laceration occurs generally as an accident of labor at full term, and especially after instrumental delivery. It cannot be doubted that lateral laceration is sometimes produced by the great size of the child's head, by the use of instruments, or by the too rapid delivery; but I have for several years regarded laceration as an accident of premature delivery. So fully am I convinced of the correctness of this statement, that I never now meet with a case of laceration that I do not enquire as to the date and number of the miscarriages. I cannot think that ruptures so extensive can occur very often at the full term of gestation; but it is easy to understand how it will occur in the early months of pregnancy by the passage of the fœtus through a cervix that is, as yet, undeveloped, and whose function it is to guard the pregnancy until the expiration of the full term of gestation. But however this may be, I am certain of the fact that lateral laceration is most commonly caused by abortion—by the premature passage of the fœtus through the unprepared or undeveloped cervix.

Dr. Emmet, in his last article, in describing the minute details of the operation, urges, as a condition of success, that the opposite edges of the laceration shall be quite thoroughly pared or cut away. This must, in many cases, produce, when the wound has healed, great conicalness of the cervix. My experience with these cases does not enable me to speak with positiveness as to the effect that this altered state of the cervix will exercise in preventing future conceptions; but knowing how frequently sterility is due to a conical cervix, I suspect that pregnancy is not common after this operation. Although this operation has been performed many hundred times in the city of New York alone, I have seen no statistics on this point. I will suggest further as my belief, that should a pregnancy occur, the cervix, should much of its substance have been removed, could hardly escape aceration at the time of delivery.

W. H. NEWMAN, M.D.

